# Differential activation of amygdala *Arc* expression by positive and negatively valenced emotional learning conditions

**DOI:** 10.3389/fnbeh.2013.00191

**Published:** 2013-12-05

**Authors:** Erica J. Young, Cedric L. Williams

**Affiliations:** Neuroscience and Behavior Graduate Program, Department of Psychology, University of VirginiaCharlottesville, VA, USA

**Keywords:** emotional arousal, memory modulation, amygdala, *Arc* expression, brain asymmetry, amygdala lateralization

## Abstract

Norepinephrine is released in the amygdala following negatively arousing learning conditions. This event initiates a cascade of changes including the transcription of activity-regulated cytoskeleton-associated protein (*Arc*) expression, an early-immediate gene associated with memory encoding. Recent evidence suggests that the valence of emotionally laden encounters may generate lateralized, as opposed to symmetric release of this transmitter in the right or left amygdala. It is currently not clear if valence-induced patterns of selective norepinephrine output across hemispheres are also reproduced in downstream pathways of cellular signaling necessary for memory formation. This question was addressed by determining if *Arc* expression is differentially distributed across the right and left amygdala following exposure to positively or negatively valenced learning conditions respectively. Male Sprague Dawley rats were randomly assigned to groups exposed to the Homecage only, five auditory tones only, or five auditory tones paired with footshock (0.35 mA) during Pavlovian fear conditioning. Western blot analysis revealed that *Arc* expression in the right amygdala was elevated significantly above that observed in the left amygdala 60 and 90 min following fear conditioning. Similarly, subjects exposed to a negatively valenced outcome consisting of an unexpected reduction in food rewards showed a greater level of *Arc* expression in only the right, but not left basolateral amygdala. Presenting a positively valenced event involving an unexpected increase in food reward magnitude following bar pressing, resulted in significantly greater *Arc* expression in the left, but not right basolateral amygdala (*p* < 0.01). These findings indicate that the valence of emotionally arousing learning conditions is reflected at later stages of synaptic plasticity involving the transcription of immediate early genes such as *Arc*.

## Introduction

Converging evidence suggests that the left and right amygdala are preferentially activated during the encoding of emotional events containing positive or negatively valenced stimuli. Molecular markers and immediate-early gene expression induced by new types of learning conditions reflect similar asymmetric patterns of activation. For example, elevated levels of protein kinase C (PKC) or cyclic AMP response element binding protein (CREB) are integral components of the molecular cascades that convert new information from short, to long term memory (Zhou et al., 2009; de Oliveira Coelho et al., 2013; Pinho et al., 2013). Surprisingly both proteins are upregulated in the right, but not left amygdala after exposure to a tone previously paired with an aversive experience such as footshock during fear conditioning training or in response to exposure to a threatening predator (Blundell and Adamec, 2007; Orman and Stewart, 2007). Another molecular marker required for regulating neural plasticity in response to inflammatory pain is extracellular signal-regulated kinase (ERK) signaling in the amygdala. Inflammation-induced mechanical sensitivity is reduced by blocking right amygdala ERK activation, regardless of the side of the peripheral injury (Carrasquillo and Gereau, 2008). Furthermore, expression of the immediate-early gene *c-fos* is elevated in the right amygdala following reexposure to the fear conditioning context. This finding demonstrates that the responsiveness of amygdala neurons are lateralized even when the organism re-experiences stimuli with a negative valence (Sciclli et al., 2004) and provides additional evidence that unpleasant or aversive events produce asymmetric changes within the amygdala that are expressed at a molecular level.

Norepinephrine released following experimental manipulations facilitates new learning and improves later retention or recall by upregulating signaling cascades leading to *Arc* dependent actin rearrangement necessary for long-term potentiation (LTP) in the hippocampus (Hou et al., 2009) as well as important cellular changes within the amygdala (Liu et al., 2012) and cingulate cortex (Holloway-Erickson et al., 2012). These findings illustrate the important contribution of norepinephrine release in the basolateral amygdala during encoding by influence on intracellular events associated with memory formation. When norepinephrine binds to post synaptic β-noradrenergic receptors it initiates cAMP formation. Elevating cAMP levels leads to the activation of cAMP dependent kinases, such as MAPK, PKA and PKC, which in turn phosphorylates CREB. Phosphorylated CREB is a transcription factor that upregulates gene transcription, including immediate-early genes such as *c-fos* and *Arc* (Davies et al., 2004; Blundell and Adamec, 2007; Orman and Stewart, 2007; Canal et al., 2008). Expression of *Arc* protein is induced by strong synaptic activation and is quickly transported to the active dendrites to participate in synaptic remodeling during associative learning and LTP (Bramham et al., 2008; Miyashita et al., 2008). Stabilization of actin molecules by *Arc* protein allows active synapses that represent features of the new event in the amygdala or hippocampus to be bound and associated together (Bramham et al., 2010). If valence dependent asymmetric release of norepinephrine is a catalyst for stimulating cellular activity, then immediate-early gene expression of activity-regulated cytoskeleton-associated (*Arc*) protein should also be disproportionally upregulated in either the right or left basolateral amygdala depending on the valence of the learning condition.

In light of these findings, these studies investigated whether exposure to stimuli of opposing valence differentially influences *Arc* expression in the right or left basolateral amygdala. Experiment 1 utilized western blots to assess *Arc* expression in the right and left amygdala at different time points following presentation of a negatively valenced stimulus consisting of footshock during Pavlovian fear conditioning. Experiments 2 and 3 expanded these finding by assessing whether training with appetitive stimuli in an operant learning task produces different patterns of *Arc* expression across the left or right basolateral amygdala following unexpected positive or negative consequences to barpressing.

## Methods

### Subjects

Sixty-two male Sprague-Dawley rats (275–300 g) obtained from Charles River Laboratories (Wilmington, MA) were used in Experiments 1 (*n* = 25), 2 (*n* = 15) and 3 (*n* = 22). Rats were individually housed in plastic cages and maintained on a standard 12:12 h light-dark cycle with lights on at 7:00 am. Food and water were available *ad libitum* during the 7 days undisturbed adaptation period to the vivarium. All experiments were conducted in accordance to the policies and guidelines of the University of Virginia's Animal Care and Use Committee.

### Behavioral procedures

#### Behavioral apparatus

The apparatus used for Pavlovian Fear conditioning and the Operant task consisted of a Coulbourn (Allentown, PA) behavioral chamber (12″W × 10″D × 12″H, Model #: H13–16) that was enclosed in a larger sound-attenuating box (28″W × 16″D × 16″H). The walls of the chamber were constructed of clear plastic with stainless steel sides and a removable stainless steel grid floor. The conditioning chambers were cleaned with a 10% alcohol solution after training.

### Fear conditioning

Rats were transported from the vivarium to the lab 4 h prior to training. The rats were first habituated to the conditioning chamber with 5 min of free exploration. Twenty-four hours later, they were placed in the training context for a 3 min baseline followed by a 30 s tone (5 kHz, 75 db) conditioned stimulus (CS) that co-terminated with a 1 s, 0.35 mA foot shock unconditioned stimulus (US). A 60 s inter-trial interval (ITI) separated a foot-shock from the presentation of the next tone. Conditioning consisted of five (CS) tone- (US) shock pairings. Freezing behavior was defined as an absence of movement except respiratory function (Blanchard and Blanchard, 1972) and was recorded with a Coulbourn (Allentown, PA), infrared activity monitor (Model #: H24–61) that automatically samples movement every 400 ms. A customized program developed by Coulbourn was used to convert recordings of the absence of movement into a Microsoft Excel macro that calculated the percent of this measure during each of the five, 30 s periods of tone presentation.

### Operant task

#### Training

All subjects were placed on a weight maintenance schedule 7 days prior to training (i.e., a 15% reduction in body weight) and remained on this schedule throughout the experiment. The animals were initially shaped to lever press for food rewards consisting of 45 mg sucrose pellets. They were then trained on a Fixed Ratio 5 schedule (FR5) over the course of 10 days. A light signaled the start of each trial and the animals were required to make five lever presses to initiate delivery of sucrose pellets to the food cup. Head pokes into the food cup interrupted an infra-red beam that turned the light off and signaled the end of each trial. There was an ITI of 30 s between each of the 10 daily trials.

#### Shift in reward expectations

Subjects were divided into two groups during the initial 10 days of training. One group received (10) sucrose pellets after each FR5 schedule as opposed to the second group that was reinforced with only (1) pellet after the five lever presses. After 10 days of training with the (1) or (10) pellet food reward, half of the animals from these two groups were randomly assigned to a *Down-shift* or *Up-shift* group that experienced an unexpected reduction or increase in food rewards respectively.

For instance, one-half of the rats originally rewarded with (10) pellets were *Down-shifted* on Day 11 and given only (1) pellet for each FR5 trial. The *Down-shift* in reward quantity elicits a negative psychological state that is reflected by a reduction in approach behavior toward the food cup and a level of frustration manifested by increased responding on the lever (Crespi, 1942; Goldman et al., 1973). Subjects in this condition are referred to as (Group 10-1) to denote the difference in reward quantity prior to and after the shift. Accordingly, subjects that continued to receive the same level of food rewards are labeled as (i.e., Group 10-10).

One-half of the remaining subjects that initially received (1) pellet following each FR5 trial were *Up-shifted* on Day 11 and rewarded with (10) sucrose pellets per trial to create a positively valenced outcome. Induction of positive affect was evidenced by increased approach behavior toward the food cup and this change was quantified by recording differences in number of “nose pokes” between Day 10 and 11 to interrupt the infra-red beam in the recessed food cup. Subjects in the *Up-shift* group are referred to as (Group 1-10) while the remaining one-half of subjects that did not experience a change in reward quantity after the shift are labeled as (i.e., Group 1-1).

### Statistical analysis

Behavioral measures from the fear-conditioning task were expressed as the mean percentage of time ± standard errors (SE) rats spend immobile during the presentation of the three retention test tones. Between-group comparisons for freezing behavior measured during retention testing was made with a factorial and repeated measures analysis of variance (ANOVAs) followed by Fisher's *post-hoc* tests. Differences less than *p* = 0.05 were considered statistically significant.

In the operant task, between-group comparisons for total number of lever presses and nose pokes made during the session on day 10 and *shift* day were made with a factorial two-way analysis of variance (ANOVA) followed by Fisher's *post-hoc* tests. Between-group and within-group comparisons for changes in lever presses and nose poking across day 10 and *shift* day were made with a repeated measures two-way analysis of variance followed by Fisher's *post-hoc* tests. A repeated measures ANOVA was also used for between-group comparisons of trial-by-trial changes in lever pressing and nose poking on each day. Differences less than *p* = 0.05 were considered statistically significant.

### Western blotting

Thirty minutes, 1 h or 90 min following completion of Pavlovian fear conditioning animals were briefly sedated with isoflorine gas and decapitated. The brains were quickly removed and placed in ice cold 0.9% saline for a few minutes. Three 2 μm slices were taken per animals and the amygdala in each hemisphere was punched with 1 μm diameter punch (Fine Tools). Punches from the right and the left amygdala for each animal were placed in separate vials containing 100 μl of RIPA buffer and 1 μl of protease inhibitor. Samples were vortexed, placed on ice for 30 min and then centrifuged at 21000 rcf for 10 min at 4°C. Total protein concentration contained in the right and left amygdala homogenate of each animal was determined using a spectrophotometer (absorbance set at 562 mm) and Micro BCA protein assay kit (Thermo scientific). Approximately 5.5 μg of protein from the left and right amygdala of each animal was combined with *Laemlli* 2 × and 5% β-mercaptoethanol and boiled at 95°C for 4 min. The protein solution was loaded into a separate well and then ran on 10% Tris-HCl gel (Bio-Rad). MagicMark (Invitrogen) was run on all of the gels to determine molecular weight for each immunoreactive band. Proteins were transferred from the gel to a nitrocellulose membrane using a semi-dry transfer cell (Bio-Rad). Membranes were blocked overnight at 4°C in a blocking buffer composed of 5% membrane blocking agent (GE Healthcare) in Tris buffered saline (TBS) and 0.1% 20-Tween (TBS-Tween). Prior to antibody incubation, membranes underwent several washes in TBS-Tween for a total of 30 min. During the first round of probing, the primary antibody was anti-Arc diluted in TBS-Tween (rabbit polyclonal; 1:3000, Synaptic Systems) for 1 h followed by an additional 30 min of washes. The membrane was then incubated for an hour with the secondary HRP-linked antibody (goat anti-rabbit; 1:3000, Millipore). Following an additional 30 min wash, chemiluminescence (ECL Plus Western Blotting detection system; GE Healthcare) was used to detect immunoreactivity. A stripping buffer (Thermo Scientific) was then applied to the membranes for 15 min. Membranes underwent five, 10-min washes in TBS-Tween. The membranes were then blocked for 1 h at room temperature in the blocking buffer mentioned previously. The second round of probing consisted of incubation in the primary antibody anti-β actin diluted in the blocking buffer (mouse; 1:10,000, Sigma) for an hour and in secondary HRP-linked antibody (goat anti-mouse; 1:80,000, Sigma). Washes and chemiluminescence were conducted in the same manner as during the first round of probing. Densitometric quantification was conducted by scanning the films and analyzed band density with Nikon Imaging Software.

### Immunofluorescence

#### Extraction procedures

One hour after completion of Pavlovian fear conditioning or the operant task, animals were anesthetized with pentobarbital. Homecage animals were brought to the lab and anesthetized with pentobarbital. All animals were then perfused transcardially with a 0.9% saline solution followed by a mixture of 4% paraformaldehyde for 5–10 min. The brains were removed and submerged in a 4% paraformaldehyde solution for 24 h, placed in a 30% sucrose solution for 48 h, dissected on a vibratome at a thickness of 50 μm and stored in phosphate buffered saline (PBS) containing 0.1 M sodium azide at 4°C.

#### Immunofluorescence procedures

Tissue samples were treated with two different fluorescence dyes to first, visualize *Arc* expression and second, to visualize cell bodies within the amygdala. The samples were incubated in an ice cold 1:1 ratio of acetone: methanol mixture for 10 min. After gentle rinsing in 2 × SSC for 5 min, the tissue was incubated for 15 min to reduce nonspecific interactions. The tissue was rinsed again in 2 × SSC for 5 min and then quenched in enogenous peroxidase for 15 min. Following thorough washes in 2 × SSC, the tissue was incubated in TSA blocking buffer for an hour and then with the primary anti-*Arc* antibody (1:1,000 dilution, SySy, Gottingen, Germany), for 24 h at 4°C. The next day after more thorough washes in 2 × SSC, the tissue was incubated in biotinilated secondary anti-rabbit biotin antibody (1:500 dilution, Vector Laboratories, Burlingame, CA) for 24 h at 4°C. The tissue was then rinsed in 2 × SSC, incubated in ABC Elite Reagent for 5 min, washed an incubated in Cyanine 3 tyramide (Cy3) reagent for 45 min (1:50 dilution, Perkin-Elmer, Waltham, MA) and dapi (1:500 dilution, Invitrogen, Carlsbad, CA) for 15 min. The samples were then washed several times in 2 × SSC tween and TBS in between the Cy3 reagent and dapi staining. Prior to mounting, the tissue was gently rinsed in TBS for 10 min.

#### Quantification procedures

**Figure 1:**
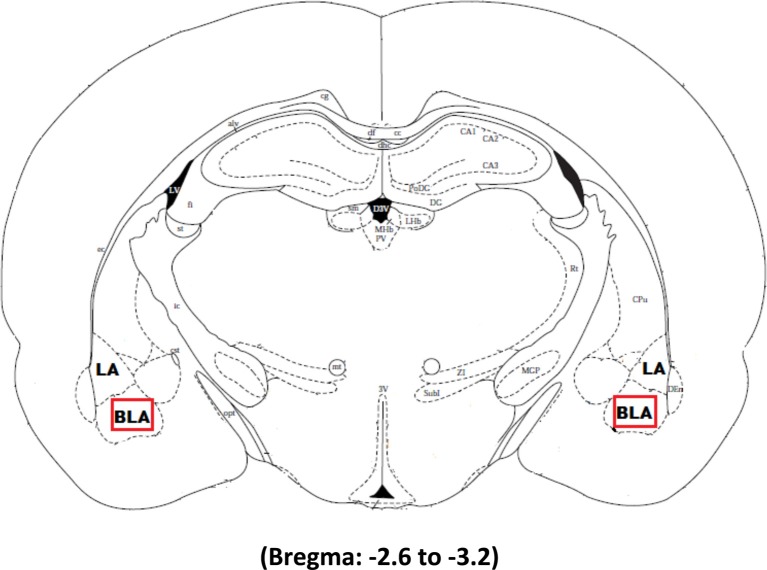
**The boxed square represents the area of interest between −2.6 and −3.2 from Bregma selected for quantification of *Arc* signal in the right and left basolateral amygdala after Pavlovian Fear Conditioning (Experiments 1 and 2) or Appetitive Training with positive or negative reinforcement contingencies (Experiment 3)**.

The tissue was imaged using Nikon imaging software at a magnification of 40×. Three slices per animal ranging from −2.6 to −3.2 from bregma were chosen for imaging. Figure 1 illustrates the area of interest in the most medial aspect of the right and left basolateral amygdala selected for imaging in Experiments 2 and 3 whereas the whole amygdala was selected for imaging in Experiment 1. *Arc* expression was analyzed using 1 μm *z* stacks with 30 steps at an exposure time set to 30 ms. The median and 2 standard deviations above the mean was determined for each image and then averaged together for each animal. The intensity threshold was individually set for each animal to be 2 standard deviations above their mean signal intensity. The size threshold was set to be the same size as a dapi cell. A macro was written to count every signal above the intensity and size threshold. Expression was quantified as the number of Cy3 labeled cells in the area of interest and a marco counted every dapi cell contained within this region to determine the percentage of cells expressing *Arc*.

## Results

### Experiment 1

#### Behavioral data

**Figure 2:**
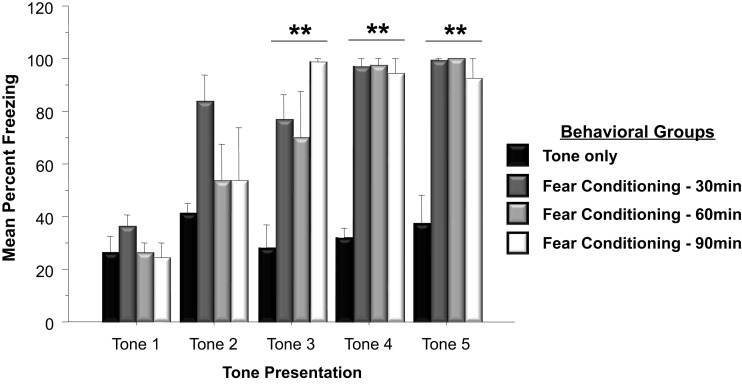
**Mean (+SE) percentage of time freezing during Pavlovian conditioning with a 30 s tone CS that coterminated with a 1 s 35 mA footshock**. Subjects in the Fear Conditioning group displayed significantly more freezing during the final three tone presentations than controls in the Tone Only Group (Fear Conditioning 30 min vs. Tone Only, *p* < 0.01; Fear Conditioning 60 min vs. Tone Only, *p* < 0.01; Fear Conditioning 90 min vs. Tone Only, *p* < 0.01). ^**^p < 0.01.

Western blots were run to analyze *Arc* protein levels in animals that had previously undergone Pavlovian fear conditioning. *Arc* protein levels associated with fear conditioning were examined 30, 60, and 90 min following training. Protein levels of animals subjected to negatively valenced stimuli during Pavlovian conditioning was compared to controls that experienced tone presentations in the absence of footshock or controls that remained within their homecage. As depicted in Figure 2, a two-way ANOVA on the behavioral data indicated that animals trained in the fear conditioning task froze significantly more than controls that only experienced five tone presentations [*F*_(3, 19)_ = 32.0, *p* < 0.01]. Comparison of freezing levels during the fifth tone presentation using a Factorial analysis revealed that animals that underwent fear conditioning froze significantly more than animals that only experienced the tone presentations (*Tone Only* vs. *Fear Conditioning 30 min*, *p* < 0.01; *Tone Only* vs. *Fear Conditioning 60 min*, *p* < 0.01; *Tone Only* vs. *Fear Conditioning 90 min*, *p* < 0.01).

#### Western blot data

**Figure 3:**
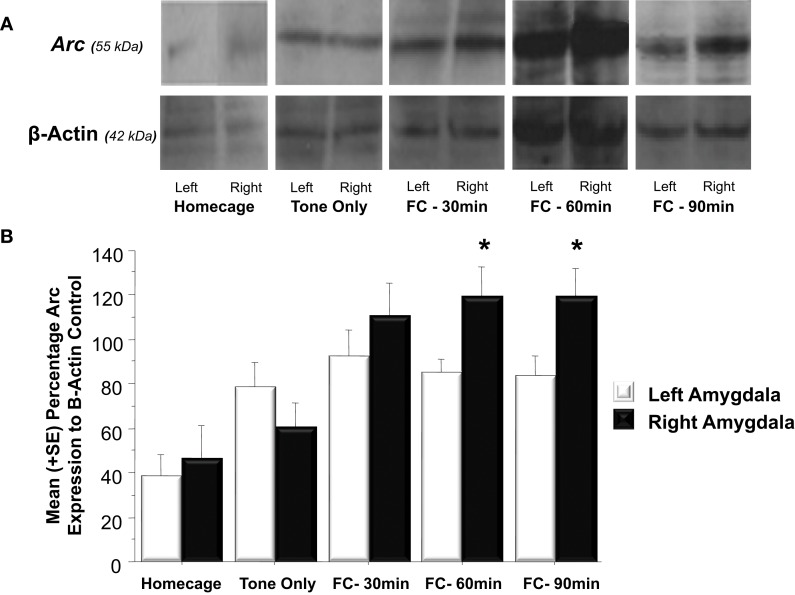
**(A)** Western blot analysis of left and right amygdala homogenates of Homecage controls, Tone only controls and animals that received Pavlovian fear conditioning with five tone-shock pairings 30, 60, or 90 min prior to sacrifice and brain extraction. **(B)** Western blot quantification of *Arc* expression in the left and right amygdala of the three groups described in **(A)**. Fear conditioning produced a significant increase in *Arc* expression relative to the change produced by resting in the homecage or following tone presentation (FC 30 min vs. Tone Only, *p* < 0.01, FC 60 min vs. Tone Only, *p* < 0.01, FC 90 min vs. Tone Only, *p* < 0.01). *Arc* levels measured in the left and right amygdala did not differ in Homecage controls, Tone Only or the Fear Conditioning 30 min group. *Arc* expression was significantly elevated in the right amygdala compared to the left at 60 and 90 min following fear conditioning (FC 60 min: right vs. left, *p* < 0.05; FC 90 min: right vs. left, *p* < 0.05). ^*^p < 0.05.

As illustrated in Figure 3A, fear conditioning significantly affected *Arc* expression in the right but not left amygdala 60 and 90 min following training [*F*_(1, 24)_ = 4.3, *p* < 0.05]. Further analysis (Figure 3B) revealed that experiencing either tone presentations or fear conditioning elicited greater *Arc* expression compared to levels measured in homecage control animals (Tone Only vs. Homecage, *p* < 0.05; Fear Conditioning 30 min vs. Homecage, *p* < 0.01; Fear Conditioning 60 min vs. Homecage, *p* < 0.01; Fear Conditioning 90 min vs. Homecage, *p* < 0.01). Furthermore, levels of *Arc* expression in fear conditioned animals was greater than levels sampled from animals presented with only the tone (Fear Conditioning 30 min vs. Tone Only, *p* < 0.01; Fear Conditioning 60 min vs. Tone Only, *p* < 0.01; Fear Conditioning 90 min vs. Tone Only, *p* < 0.01). Factorial analysis showed that *Arc* is not expressed at similar levels in the left and right amygdala following training. Thirty minutes following training, *Arc* is expressed at similar levels in both the right and left amygdala (*p* = *ns*). However, 60 min following training there was significantly more *Arc* protein in the right amygdala compared to the left (*p* < 0.05). *Arc* expression in the right amygdala was still elevated compared to the left 90 min following training (*p* < 0.05). These findings indicate that Pavlovian fear conditioning elicits asymmetric expression of *Arc* 60 min following training which remains elevated for an additional 30 min.

### Experiment 2

#### Behavioral data

**Figure 4:**
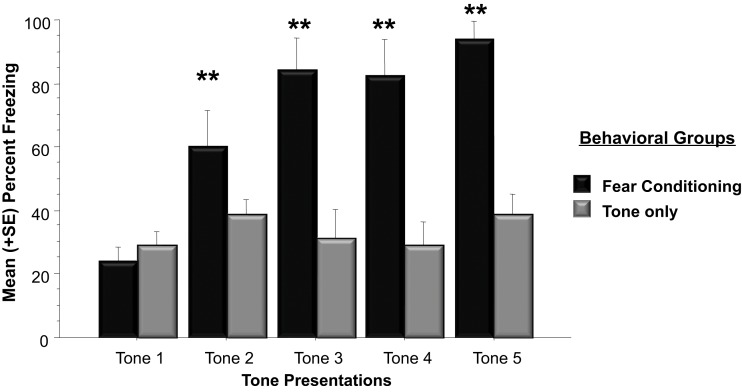
**Mean (+SE) percentage of time spent freezing during training to each tone presentation**. Animals utilized for immunohistochemistry underwent either five tone presentations or fear conditioning consisting of five tone-shock pairs. Animals that received a footshock coterminating with the tone presentation froze significantly more during Tones 2–5 (Tone 2: Fear Conditioning vs. Tone Only, *p* < 0.01; Tone 3: Fear Conditioning vs. Tone Only, *p* < 0.01; Tone 4: Fear Conditioning vs. Tone Only, *p* < 0.01; Tone 5: Fear Conditioning vs. Tone Only, *p* < 0.01). ^**^p < 0.01.

The western blots conducted in Experiment 1 examined *Arc* protein levels in the whole amygdala whereas Experiment 2 examined *Arc* protein expression in the basolateral amygdala. The right and left basolateral amygdala was imaged using immunohistochemistry to determine *Arc* expression following a physically unpleasant training task, Pavlovian fear conditioning. A two-way ANOVA indicated that animals that received footshocks during training froze significantly more than animals that only experienced tone presentations [*F*_(1, 10)_ = 39.8, *p* < 0.01]. As shown in Figure 4, animals assigned to the fear conditioning group froze significantly more than animals in the Tone Only group during Tone 2 (*p* < 0.01), Tone 3 (*p* < 0.01), Tone 4 (*p* < 0.01), and Tone (*p* < 0.01).

#### Immunohistochemistry data

**Figure 5:**
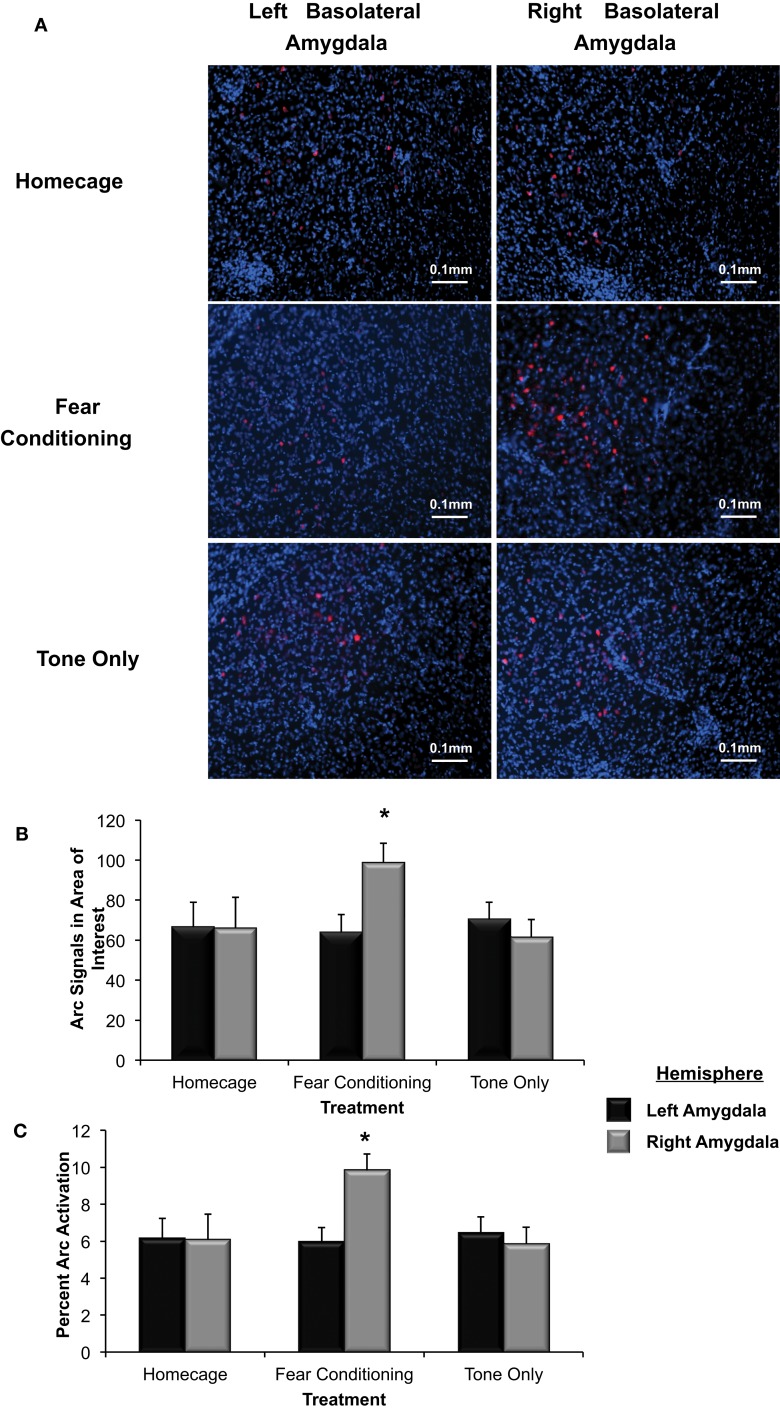
**(A)** Immunohistochemical analysis of the effect of fear conditioning on *Arc* expression in the right and left basolateral amygdala. Fear conditioning elevates *Arc* immunoreactivity in the right, but not left basolateral amygdala. **(B)** Mean (+SE) number of *Arc* signals measured in the basolateral amygdala. Tone presentation alone did not alter *Arc* expression in the basolateral amygdala compared to the signals measured in Homecage controls. Fear conditioning with five tone-shock pairings produced a significant increase in *Arc* expression that was statistically greater in the right, relative to left basolateral amygdala (^*^p < 0.05). The level of *Arc* expression in the right amygdala of the Fear Conditioning group was also significantly greater than *Arc* expression measured in the right or left amygdala of homecage controls or the Tone only controls (^*^p < 0.05). **(C)** Quantification of *Arc* expression in the amygdala reveals that the percentage of cells expressing *Arc* was only elevated in the right basolateral amygdala of animals that experienced fear conditioning when compared to Homecage controls or Tone only controls (^*^p < 0.05).

Tissue samples extracted from the right and left basolateral were processed to determine if *Arc* expression was asymmetrically distributed in response to Pavlovian fear conditioning (see Figure 5A). As shown in Figure 5B, a factorial analysis demonstrated no significant differences in *Arc* expression in the right and left basolateral amygdala of homecage controls (*p* = *ns*) or those presented with the tone only. However, right basolateral amygdala *Arc* expression in fear conditioning animals was significantly higher than levels found in the right or left amygdala of homecage controls (^*^p < 0.04 for both comparisons) as well as both hemispheres in subjects exposed to only the CS-tone (^**^p < 0.01 vs. right; ^*^p < 0.05 vs. left amygdala).

To further test *Arc* expression levels in the basolateral amygdala following a negatively valenced learning experience, the percentage of cells that displayed *Arc* signaling was determined (see Figure 5B). There was a significant overall effect of hemisphere and group on the percent of *Arc* activation [*F*_(5, 244)_ = 2.91, *p* < 0.03]. The percent of cells that displayed *Arc* signaling in animals assigned to the homecage group was not significantly different between hemispheres (*p* = *ns*). In animals that experienced fear conditioning there was a significantly higher percent of cells that displayed *Arc* signal in the right basolateral compared to the left (*p* < 0.05). As shown in Figure 5C, there were no differences in *Arc* expression between the right and left basolateral amygdala in animals assigned to the Tone Only group (*p* = *ns*). The percent of cells expressing *Arc* in the right basolateral amygdala of Fear Conditioning animals was significantly higher than levels measured in Homecage or Tone Only animals (*p* < 0.05). These finding indicate that exposure to emotionally aversive learning experiences such as Pavlovian fear conditioning induces an asymmetric increase of *Arc* expression in the right basolateral amygdala.

### Experiment 3

#### Behavioral data

**Figure 6:**
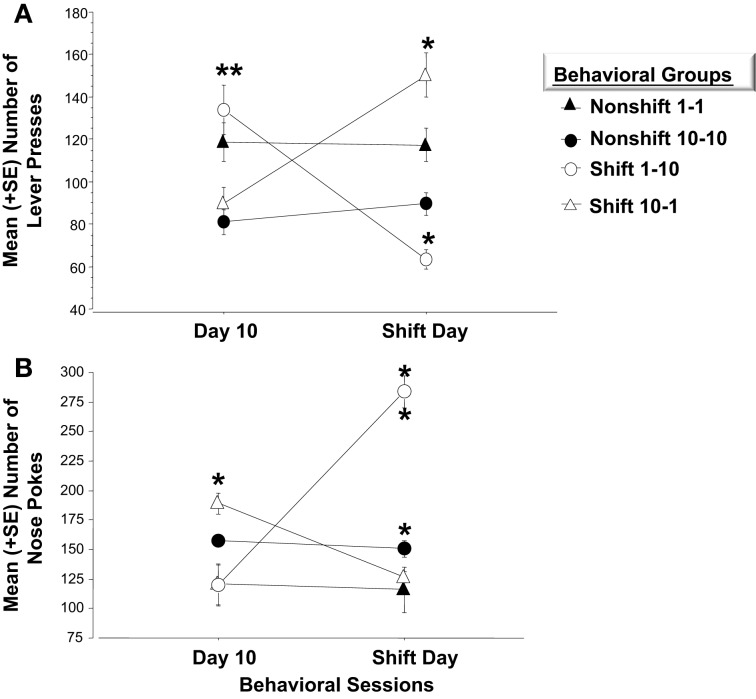
**(A)** Mean (+SE) number of lever presses on the final day of initial training and in response to the unexpected change in reward magnitude on the *Shift Day*. On Day 10, animals accustomed to only (1) sucrose pellet following each FR-5 schedule (i.e., *open circle and closed triangle*), make significantly more lever presses than those given (10) sucrose pellets for each FR-5 during the first 10 days of training, (^**^p < 0.01). Animals assigned to either of the *Nonshift* groups (i.e., *closed triangle and circle*) did not alter lever pressing behavior on the *Shift* day. A decrease in reward quantity (i.e., *Shift 10-1; open triangle*) caused a significant increase in lever press responses relative to the number recorded on Day 10 (^**^p < 0.01) before the decrease in reward magnitude. Subjects in the *Down-shift* group displayed a significantly higher level of lever presses on the *Shift* day compared to animals that received (1) sucrose reward throughout the experiment (^**^p < 0.01; *open triangle* vs. *closed triangle*). Conversely, animals that experienced the *Up-shift* in reward from (1) to (10) sucrose pellets (i.e., *open circle*) decreased lever pressing behavior on the *Shift* day compared to Day 10 (^**^p < 0.01). **(B)** Mean (+*SE*) number of nose pokes into the food hopper on the last day of training and on the *Shift* day. On Day 10, the last day of training, animals that received (10) sucrose pellet following each FR 5, nose poked into the food hopper significantly more than animals that received (1) sucrose pellets (*p* < 0.05). *Non-shift* groups received the same reward magnitude throughout the experiment and did not altered nose poke behavior on Day 11. Animals that experienced an *Up-shift* in reward magnitude from (1) to (10) sucrose pellets increased nose poke behavior following the shift compared to Day 10 (*p* < 0.01). Additionally, these animals made more nose pokes on the *Shift* day compared to animals assigned to the *Non-shift* 10-10 group (*p* < 0.01). Conversely, animals that received a *Down-shift* from (10) to (1) sucrose pellets decreased nose poke behavior compared to Day 10 behavior (*p* < 0.05). ^*^p < 0.05; ^**^p < 0.01.

Experiment 1 and 2 utilized two different molecular approaches to examine *Arc* expression following exposure to negatively valenced noxious stimuli associated with Pavlovian fear conditioning. Experiment 3 extends these findings by examining whether induction of positive affect by increasing the magnitude of expected rewards or alternatively, generating frustration by violating a subject's expectations of reinforcement may be registered by more robust patterns of *Arc* expression across the left and right amygdala respectively. Each of four groups received 10 consecutive days of training. Two of these groups were rewarded with one pellet after completing each FR schedule and the other pair was given the higher quantity of 10 pellets. Factorial analysis on mean bar presses per group revealed a significant overall effect [*F*_(3, 21)_ = 37.5, *p* < 0.01]. Fisher's *Post-hoc* analysis revealed that the magnitude of reward (1 or 10 pellets) influenced the number of lever presses. As illustrated in Figure 6A, subjects given one pellet made significantly more lever presses than those in the 10 pellet reward group (1-1 vs. 10-10, *p* < 0.01; 1-1 vs. 10-1, *p* < 0.05; 1-10 vs. 10-10, *p* < 0.01; 1-10 vs. 10-1, *p* < 0.01). However, there were no differences in lever press performance between paired groups given the same quantity of reward before the experimental shift (i.e., 10-10 vs. 10-1; *p* = *ns*; 1-1 vs. 1-10; *p* = *ns*). Reward magnitude also influenced the number of nose pokes into the food dispenser. Factorial ANOVA's run on the mean number of nose pokes during Day 10 revealed a significant overall effect [*F*_(3, 21)_ = 58.2, *p* < 0.01]. *Post-hoc* analysis revealed inverse trends to bar pressing performance. Mainly, subjects rewarded with 10 sucrose pellets made more pokes than those in the one pellet groups (Non-shift 10-10 vs. Non-shift 1-1, *p* < 0.05; Non-shift 10-10 vs. Up-shift 1-10, *p* < 0.01; Down-shift 10-1 vs. Non-shift 1-1, *p* < 0.05; Down-shift 10-1 vs. Up-shift 1-10, *p* < 0.01) (Figure 6B).

Animals in the non-shifted groups continued to receive the same quantity of rewards on the shift day as during training (i.e., 1-1 and 10-10). Thus, no changes in lever pressing were expected on the shift day. Repeated measures ANOVA verified that these groups did not differ on any of the behavior measures. However, the unexpected increases or decreases in reward quantity caused by the shift on Day 11, significantly affected lever press and nose poke behavior. A repeated measure ANOVA indicated that bar pressing performance in subjects experiencing the downshift (i.e., from 10 to 1 pellet) increased significantly on the day of the shift (*p* < 0.01) while nose poke responses decreased (*p* < 0.05). Conversely, animals assigned to the upshift group and therefore given 10 as opposed to 1 sucrose pellet displayed a significant decrease in bar pressing (*p* < 0.01) while significantly increasing nose poke behavior (*p* < 0.01). The changes in lever press performance observed in the “Frustrated” downshift group (10-1) and in the “Elated” upshift group (1-10) conformed to Crespi's (1942) finding that previous reward history and expectations of reward quantity impacts later performance when these expectations are violated.

Factorial analysis on behavior of Day 11 revealed a significant overall effect of changes in reward quantity on bar press [*F*_(3, 21)_ = 24.8, *p* < 0.01] and nose poke behavior [*F*_(3, 21)_ = 38.3, *p* < 0.01]. As shown in Figure 6A, animals assigned to the Frustrated, downshift group made more lever presses after the decrease in reward quantity than the non-shifted subjects that continued to receive 10 pellets (*p* < 0.01). They also bar pressed more than subjects assigned to receive one pellet for the duration of the study (1-1) (*p* < 0.01). Conversely, animals assigned to the upshift group made significantly fewer lever presses following the increase in reward quantity than animals assigned to the nonshift groups (1-10 vs. 10-10, *p* < 0.05; 1-10 vs. 1-1, *p* < 0.01). Additionally, as shown in Figure 6B these animals made significantly more nose pokes than animals that were rewarded with 10 sucrose pellets throughout training (*p* < 0.01).

#### Immunohistochemistry data

**Figure 7:**
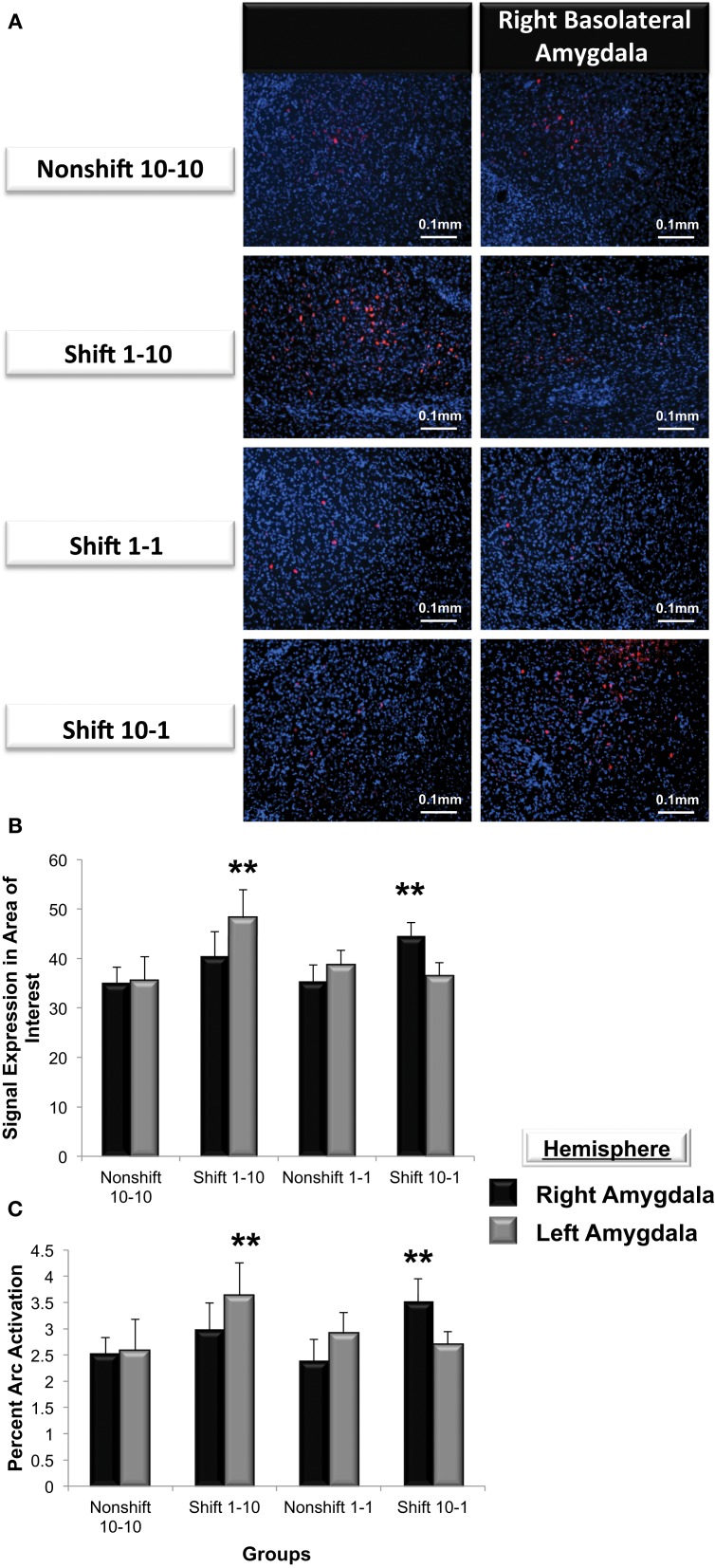
**(A)** Immunohistochemical analysis of *Arc* expression in the basolateral amygdala after an unexpected increase (*Shift* 1-10), decrease (*Shift* 10-1) or no change in reward magnitude following FR-5 lever pressing. The behavioral manipulations produced a significant change in left basolateral *Arc* expression only following an increase in reward quantity (*Shift* 1-10), whereas right basolateral *Arc* expression was elevated only by the unexpected reduction in reward quantity after the FR-5 (*Shift* 10-1). **(B)** Mean (+SE) number of *Arc* signals measured following the Shift in reward expectancies. There were no changes in *Arc* signal sampled from the amygdala of Non-shifted animals. *Arc* signals in the left basolateral amygdala were significantly greater than levels in the right (^**^p < 0.01) in subjects assigned to receive an *up-shift* in reward quantity (*Shift* 1-10). Conversely, *Arc* signals in the right basolateral amygdala were elevated compared levels sampled from left, in the group that experienced an unexpected *Down-shift* (*Shift* 10-1) in reward magnitude (^**^p < 0.01). **(C)** Quantification of the mean percentage of basolateral cells that express *Arc* following presentation of stimuli of opposing valence. Approximately 2.5% of cells in the basolateral amygdala express *Arc* in animals that received an expected reward quantity of either 10 or 1 sucrose pellets. Significantly more cells in the left, but not right basolateral amygdala expressed *Arc* following an expected *Up-shift* in reward quantity (^**^p < 0.01). Conversely, following a *Down-shift* more cells in the right basolateral amygdala expressed *Arc* compared to levels sampled in the left basolateral amygdala (^**^p < 0.01).

*Arc* expression was measured following a violation of the organism's expectation of reward quantity to determine whether these emotional reactions are reflected by asymmetric patterns of *Arc* expression in the left or right basolateral amygdala. As illustrated in Figure 7A, *Arc* expression in area of interest was elevated in the right but not left basolateral amygdala following a downshift in reward quantity (i.e., *from 10-1 food pellets*) and elevated in the left but not right following an upshift (i.e., *from 1-10 food pellets*) in reward quantity [*F*_(3, 21)_ = 3.9, *p* < 0.01]. Factorial analysis revealed that *Arc* expression measured in the right or the left basolateral amygdala in nonshift groups was not significantly different (1-1: right vs. left, *p* = *ns*; 10-10: right vs. left, *p* = *ns*). As shown in Figure 7B, animals that expected 10 sucrose pellets and were downshifted to receive only 1 pellet exhibited a greater level of *Arc* expression in the right but not left basolateral amygdala (right vs. left, *p* < 0.01). Conversely, subjects that experienced an upshift from 1 to 10 sucrose pellets there was greater *Arc* expression in the left but nor right basolateral amygdala (right vs. left, *p* < 0.01).

To validate these findings, *Arc* expression was quantified as the percentage of cells located in the area of interest expressing *Arc* (Figure 7C). The valence of a learning task selectively increased the percentage of cells expressing *Arc* in one amygdala vs. the contralateral side (*F*_(3, 21)_ = 5.2, *p* < 0.01). Factorial analysis revealed that the percent *Arc* expression measured in the right or the left basolateral amygdala in nonshift groups was not significantly different (1-1: right vs. left, *p* = *ns*; 10-10: right vs. left, *p* = *ns*). Consistent with the findings described above, the downshift in reward quantity produced a greater percent change in *Arc* expression in the right but not left basolateral amygdala (right vs. left, *p* < 0.05) whereas the positively valenced upshift led to greater levels of Arc expression in the left but not right basolateral amygdala (right vs. left, *p* < 0.01).

## Discussion

Previous reports from functional scans (Morris et al., 1999; Zalla et al., 2000; Hamann et al., 2002; Etkin et al., 2004), lesions (Blundell and Adamec, 2007; Orman and Stewart, 2007; Carrasquillo and Gereau, 2008), and electrophysiology studies (Lanteaume et al., 2007; Ji and Neugebauer, 2009) indicate that the amygdala encodes emotionally arousing events differentially, depending on the valence of the stimuli Additionally, a novel finding to emerge from our lab is that norepinephrine released in the basolateral amygdala encodes negatively or positively arousing learning tasks through asymmetric norepinephrine release in right or left basolateral amygdala, respectively (Young and Williams, 2010). Furthermore, there is evidence that enhancing asymmetric norepinephrine activity in the right, but not left basolateral amygdala potentiates memory formation for negatively arousing events (LaLumiere and McGaugh, 2005). However, whether asymmetric neurotransmitter activity in the amygdala influences molecular events associated with memory formation has not been investigated. The current studies were developed to address this shortcoming in the literature by assessing whether *Arc* is selectively expressed in either the right and left basolateral amygdala following exposure to either negatively or positively arousing learning conditions.

To assess valence dependent expression of *Arc* in the amygdala, it was first necessary to determine if the valence of a learning condition produces differential patterns of *Arc* across the right or left amygdala and then examine whether the expression fluctuates during the time period this gene plays an active role in memory formation. Experiment 1 was instrumental in achieving this objective by utilizing western blot analysis of *Arc* expression in the right and left amygdala of naïve, non-aroused and negatively aroused animals. For example, experiencing either the non-arousing five tone presentations or the negatively arousing fear conditioning increased overall *Arc* expression by 162 and 237%, respectively, compared to levels measured in naïve Homecage animals. Furthermore, experiencing fear conditioning elicited significantly greater overall *Arc* expression in the amygdala compared to animals that were only presented with the auditory tones. Further analysis comparing levels of *Arc* in the right and left amygdala revealed that there was no significant different between *Arc* levels sampled from the right and left amygdala in Homecage and Tone Only animals. Interestingly asymmetric differences were only found in animals that underwent Pavlovian fear conditioning.

The second objective of Experiment 1 was to ascertain whether selective expression of *Arc* fluctuated across the three sampling periods selected at 30, 60, and 90 min following Pavlovian fear learning. For example, *Arc* levels in the right and left amygdala did not differ significantly at 30 min following Pavlovian fear learning but were elevated in the right amygdala compared to the left at 60 and 90 min following training. This result is consistent with previous findings showing that *c-fos* expression, a different immediate-early gene, is asymmetrically elevated in the right amygdala following reexposure to a negatively arousing context (Sciclli et al., 2004). Furthermore, the current findings indicate that *Arc* expression in the right amygdala is downstream of norepinephrine release in the basolateral amygdala. Fear conditioning was previously shown to significantly elevate norepinephrine release in the right basolateral amygdala compared to the left 20 min following training and to remain elevated for 40 min (Young and Williams, 2010). The findings that emerged from this study indicates that *Arc* expression in the right amygdala is significantly elevated from levels sampled from the left at 60 and 90 min following training by which time norepinephrine levels are returning to baseline amounts.

Experiment 2 extended these findings by assessing whether *Arc* expression in the basolateral amygdala is selectively elicited in the right basolateral amygdala following fear learning since norepinephrine concentrations are elevated in the right but not left basolateral following this type of training (Young and Williams, 2010) and treatments that increase activity of this transmitter in only the right basolateral are is sufficient to enhance memory (Coleman-Mesches et al., 1996; Baker and Kim, 2004; LaLumiere and McGaugh, 2005). Based on findings garnered from Experiment 1, *Arc* expression was measured in naïve animals or 60 min following either five tone presentations or fear conditioning. Fear conditioning with five tone-shock pairings produced significantly more freezing during presentation of the last four tones than that observed in subjects given tone-only presentations without footshock. The differences in freezing reflect the level of associative CS-US learning in the former group whereas the Tone Only subjects displayed minimal levels of fear to the tone since this auditory cue was not accompanied with an aversive consequence like footshock. Immunohistochemical findings from this experiment differ from finding in Experiment 1 in that tone only presentations did not significantly alter *Arc* levels in the basolateral amygdala compared to Homecage animals. This finding is likely due to the fact that the majority of tonal information is processed by the lateral, and not the basolateral amygdala. Therefore, overall *Arc* expression in the whole amygdala would reflect tone presentation whereas *Arc* expression in the basolateral amygdala would not.

Findings emerging from Experiment 2 reveal that exposure to negatively arousing learning conditions are accompanied by a lateralized increase in *Arc* expression in the right basolateral amygdala. Naïve Homecage and Tone Only animals did not demonstrate hemispheric differences in *Arc* expression whereas those subjected to tone-shock pairings during fear conditioning displayed a significantly higher level of *Arc* signals and percentage of cells expressing *Arc* in the right, but not left basolateral amygdala. Approximately, 3.6% of cells, 45 cells total, in the right basolateral amygdala expressed *Arc* following fear conditioning. The level of *Arc* expression sampled from the amygdala corresponds to levels reported previously in the dentate gyrus of the hippocampus and the basolateral amygdala. For example, approximately 2% of cells in the basolateral amygdala of Homecage controls expressed *Arc*, a number that is slightly higher than the 1.6% of cells previously reported to express *Arc* in the dentate gyrus (Small et al., 2004). While there the majority of studies examine *Arc* expression following negative arousal there is paucity of studies that examine the mechanisms that permit positively arousing stimuli to produce lateralized changes in neuronal functioning in the basolateral amygdala.

To verify if *Arc* expression in the basolateral amygdala is affected by positively or negatively valenced outcomes, an appetitive behavioral task with reinforcement contingencies of opposing valance was utilized to elicit either positive or negative arousal. Experiment 3 was instrumental in meeting this objective by including an *Up-shift* or *Down-shift* in expected reward magnitude to represent positive and negative changes in the subjects learned expectations respectively. Accordingly, these experimental manipulations produce patterns of responding characteristic of those observed following exposure to positive or negatively valenced environmental events. For example, animals subjected to the *Up-shift* in reward quantity from (1) to (10) sucrose pellets displayed a 48% reduction in lever press behavior relative to their performance on the previous non-shifted day of training. *Up-shift* animals also made significantly fewer level presses than animals that received 10 sucrose pellet throughout training. Interestingly, animals assigned to the *Up-shift* displayed a 237% increase in nose poke behavior compared to Day 10. This increase in behavior is not the result of receiving 10 sucrose pellets because *Upshift* animals made on average 133 more nose pokes on Day 11 than animals that received 10 sucrose pellets throughout training. Rather, this form of increased behavioral responding is most likely due to the induction of positive affect associated with the organism's perception of the large contrast in the magnitude of expected vs. actual rewards (Crespi, 1942). Increases in reward magnitude has been used previously to amplify positive affect as laboratory rats increase consumption through lick rate during an *Up-shift* produced by higher levels of sucrose concentration (Ainge et al., 2006; Liang et al., 2008). However, licking behavior is decreased at the beginning of a trial when animals anticipate an *Up-shift* in sucrose concentrations to occur during the latter half of the trial (Weatherly et al., 2005).

An important finding to emerge from this study is that a positively arousing learning conditioning consisting of an unexpected increase in reward quantity elicited a significant elevation in *Arc* expression in the left but not right basolateral amygdala. For example, the brains of animals surprised with an unexpected increase in reward quantity to 10 vs. 1 food reward displayed a higher percentage of cells in the left basolateral amygdala that expressed *Arc* compared to the right. This increases in *Arc* expression was also significantly greater than that observed in subjects that received the expected reward of 10 sucrose pellets throughout training. Additionally there was no difference between *Arc* levels measured in the right and left basolateral amygdala of these animals. These findings indicate there are significant asymmetric changes in the levels of *Arc* expressed in the left basolateral amygdala following exposure to a positively arousing learning event. A number of studies have examined *Arc* expression following stressful events, such as fear conditioning, conditioned place aversion or novelty (Ploski et al., 2008; Barot et al., 2009; Hou et al., 2009; Panja et al., 2009). This is the first study to investigate *Arc* expression following a rewarding experience and it indicates that a similar number of cells encode rewarding learning conditions in the left compared to *Arc* expressing cells in the right basolateral amygdala following a negatively arousing learning condition.

In contrast to the behavioral *Up-shift* findings, a reduction in the expected number of rewarding sucrose pellets produced by a *Down-shift* in expected reward quantity led to dramatic increases in lever press responding. After the *Down-shift* in reward quantity, animals increased lever pressing behavior by 169%. An indication of increased arousal following the decrease in reward quantity is that *Down-shifted* subjects made significantly more lever presses than animals that received the (1) sucrose pellet reward throughout the 10 days of training as well as on the 11 th day of the shift. This type of negatively valenced event in the face of reduced reward has been associated with the onset of frustration and is replicated in several behavioral conditions that involve a discrepancy between an organism's expectation and the actual amount of reward that it receives (Crespi, 1942; Levine et al., 1972; Goldman et al., 1973; Hatfield et al., 1996; Salinas et al., 1997). Frustration-induced increases in responding are produced by similar manipulations that violate an organism's expectations by reducing the expected magnitude of a reward (Amsel and Roussel, 1952; Corr, 2002).

The present findings expand previous reports by revealing that *Arc* expression is also elevated in the right basolateral amygdala following a behavioral condition that produces frustration. Sixty minutes following unexpected decrease from 10 to 1 sucrose reward pellets there was significantly more signals in the right basolateral amygdala compared to signals measured in the left. Additionally, there were a greater percentage of cells in the right basolateral amygdala that expressed *Arc* compared to the percentage of cells expressing *Arc* in the left basolateral amygdala. Approximately 45 cells in the area of interest in the right basolateral amygdala express *Arc* which is reasonable if 80 cells in the whole basolateral amygdala were labeled for *Arc* expression following fear conditioning (Ploski et al., 2008). Additionally, the level of *Arc* expression in the right basolateral amygdala following the *Down-shift* was similar to levels measured in the right basolateral amygdala following fear conditioning. These findings are not the result of reward magnitude because animals that received the expected 1 sucrose reward displayed significantly lower bilateral levels of *Arc* expression. Taken together, the present findings expand upon the current knowledge of *Arc* activity by indicating that stimuli of different valences are encoded asymmetrically in either the right or left basolateral amygdala for negatively or positively arousing learning conditions.

### Conflict of interest statement

The authors declare that the research was conducted in the absence of any commercial or financial relationships that could be construed as a potential conflict of interest.
